# A topic models analysis of the news coverage of the Omicron variant in the United Kingdom press

**DOI:** 10.1186/s12889-023-16444-7

**Published:** 2023-08-09

**Authors:** Eric Mayor, Alessandro Miani

**Affiliations:** 1 Department of Psychology, University of Basel, Missionsstrasse 62a, 4055 Basel, Switzerland; 2 University of Neuchâtel, Neuchâtel, Switzerland

**Keywords:** COVID-19, Omicron, Social representations, Public understanding, Media analysis

## Abstract

**Background:**

The COVID-19 pandemic has caused numerous casualties, overloaded hospitals, reduced the wellbeing of many and had a substantial negative economic impact globally. As the population of the United Kingdom was preparing for recovery, the uncertainty relating to the discovery of the new Omicron variant on November 24 2021 threatened those plans. There was thus an important need for sensemaking, which could be provided, partly, through diffusion of information in the press, which we here examine.

**Method:**

We used topic modeling, to extract 50 topics from close to 1,500 UK press articles published during a period of approximately one month from the appearance of Omicron. We performed ANOVAs in order to compare topics between full weeks, starting on week 48 of 2021.

**Results:**

The three topics documenting the new variant (Omicron origins, Virus mutations, News of a new variant) as well as mentions of vaccination excluding booster, Scotlands First minister statement (Communications) travel bans and mask wearing (Restrictions) and the impact of market and investing (Domains and events) decreased through time (all *p*s < .01). Some topics featured lower representation at week two or three with higher values before and after: Government’s Scientific Advisory Group for Emergencies recommendations (Communications), Situation in the US, Situation in Europe (Other countries and regions), all *p*s < .01. Several topics referring to symptoms and cases—e.g., rises of infections, hospitalisations, the pandemic the holidays, mild symptoms and care; restrictions and measures—e.g., financial help, Christmas and Plan B, restrictions and New Year; and domains of consequences and events—e.g., such as politics, NHS and patients, retail sales and airlines, featured increasing representation, (all *p*s < .01). Other topics featured less regular or non-significant patterns. Conclusion. Changes in sensemaking in the press closely matched the changes in the official discourse relating to Omicron and reflects the trajectory of the infection and its local consequences.

At the end of 2019, the outbreak of the new SARS-Cov2 virus, which was broadly labelled “the coronavirus”, in the province of Wuhan, China, quickly caused concern in most countries around the world. A few weeks later, on March 11 2020, the World Health Organization declared the pandemic stage of the coronavirus. Several waves of infections, driven by different variants of the virus, occurred in 2020 and 2021, causing numerous fatalities, high hospitalization rates, national and regional lockdowns, broad negative economic consequences, and reduced psychological wellbeing [1–4].

But the escalation of infections had not yet been as steep as that due to the Omicron variant, which was discovered on November 24 2021 [5]. The high number of mutations of the variant—amounting to 50, the highest observed—immediately alerted the scientific community [6]. Several scientific articles reported that Omicron could cause rather frequent reinfections and escape vaccines [7, 8]. It was also postulated that infection with Omicron could potentially lead to less severe symptomatology compared with the delta variant [9, 10]. As of December 18 2021, the variant was already present in 89 countries and was spreading fast [11].

Tackling Omicron was publicly mentioned as a public health priority in the United Kingdom rather quickly. During the weeks following the first Omicron cases found in the region (November 27 2021 [12];), officials mentioned cases doubling every 1.5 to 3 days depending on the period and the area, forecasting that Omicron would be the dominant variant by the end of the year. These concerns were broadly relayed by the press.

The public understanding of emerging diseases has been investigated in various studies (e.g. [13–16]), drawing upon social representations theory notably. Social representations theory focuses on how lay people make sense of new objects of knowledge such as ideas and events [17]. When relying upon textual productions (originating notably from individuals or journalists), such studies typically investigate the themes emerging from these productions though manual coding. Here we use automated analyses for the aim of exploring the dynamic nature of the public understanding of the Omicron variant. We explore which themes were present in close to 1,500 journalistic pieces related to Omicron in the UK from the days following its discovery to the end of 2021, and how these themes evolved through that period.

## Emerging infectious diseases and the news

Social representation theory is interested in the process of collective sensemaking about unfamiliar objects (e.g., ideas, events) and how these representations are shared in society. Through the processes of objectivation and anchoring, individuals perform a selection of information regarding a new or evolving object to make it concrete and congruent with familiar objects [17]. In other words, this “involves the elaboration of the tension between continuity and innovation” ([18], p. 57) in public understanding. The diffusion of scientific ideas, in the press notably, provides the information the individuals need for these processes to occur, and allows for common understanding to emerge [16, 19, 20].

The study of press coverage regarding emerging infectious diseases is informative of the evolution of sensemaking [16, 21], notably because the media can influence what people think on new matters and their response to risk [15]. Making sense of new and evolving objects is a necessary and evolving process for the adequate coping by individuals and efficient action by collectivities [22, 23].

Past research has examined sensemaking of different emerging infectious diseases and, at times, the evolution of this understanding in the press. For instance [16], have qualitatively investigated the themes of UK press articles reporting on an outbreak of Ebola in African countries. The authors also conducted interviews and compared the themes in both corpora. They found that *othering* (making the issue foreign to the UK population) was a reassurance mechanism employed by the press, which was also present in interviews. The authors also found *threat* as another theme in both press articles (particularly appearing in tabloids compared to broadsheet newspapers) and interviews, with mentions of harmful symptoms and potential risks in the UK. Finally, whether Ebola was under *control* was also a theme that was shared in both corpora (e.g., infections with Ebola being largely limited to African countries). [14] qualitatively examines the evolution of the tone and content of press articles relating to the emergence of SARS (March 2003) spanning over 4 weeks. The findings show that at the beginning, the articles focus on the potential of SARS to become a pandemic, and on discussions on the origins of the virus. Later on, the press focused on the responsibility for the management of the outbreak and its potential economic consequences. More recently [21], examined the evolution of the mentions of collectives (3 waves of data collection) in relation to the H1N1 *Swine flu* pandemic in the Swiss press and in interviews, and their associated roles of villains, victims and heroes in the interviews. They notably report some similarity between the mentions of collectives and their evolution in the press and in interviews.

## COVID-19 and the news

[24] have shown that there can be a correspondence between sentiment in the news on COVID-19 and the cases that are observed with varying effects between countries [25]. have used automated methods to detect conspiracy theories in the news and social media [13]. have investigated the dynamics of the early understanding of COVID-19 in the press and institutional discourse in Italy. They propose a narrative interpretation of the information delivered to the public, focusing on different phases such as a) the progression in the diffusion of information on the coronavirus, initially construed as a distant and a threat in other countries, and then a threat that has reached 'home' as the first cases appeared in Italy; b) communication related to the lockdown measures, presented as a requirement in a war against an invisible enemy (war metaphors); c) the transition to more normal circumstances, with disagreement between experts regarding the end of some restrictions, potentially leading to uncertainty in the public. [26] examine the social representation of “social distancing”—an unfamiliar concept for the public at the beginning of the pandemic—in UK newspapers. They find that social distancing was construed initially as a potential threat to normal life—as the possibility of the measures were only mentioned in the PM's address and the press (March 3–4 2020), then as a present and necessary threat to social life—as the measures were put in place (March 16–17 2020). The journalistic stance appeared to switch to moralization and blame (e.g., “frantic families”) as people disregarded social distancing principles, e.g., for stocking provisions, after the announcement of the lockdown (March 23–24 2020). The press then continued blaming the flouters of lockdown measures and praised those compliant with the guidelines, of which the necessity was emphasized (April 8–9 2020). Thomas and colleagues [27] focused on responsibility portrayed in articles of two Australian newspapers at the beginning of the COVID-19 pandemic (from January 20 to March 31 2020). The authors report on the evolution of associated themes, grouped in 4 frames (Causal attribution—e.g., spread of coronavirus, high density living; Moral evaluation—e.g., government making good decisions, panic selling on markets; Problem definition—e.g., economic impact of border closures, economic growth vs public health; and Treatment recommendations, e.g., cancel flights, social isolation) and 3 areas of issues (medical, behavioral and societal). They find that most frequent themes (e.g., panic buying, school closures, job losses) are in the societal area throughout the considered period, and a higher frequency of problem definition themes in the first 7 weeks.

Ittefaq and colleagues focused on the role of newspapers in propagating or attenuating the racialization of the pandemic in their articles (e.g., China virus), comparing different countries (China, the US, and the UK) [28]. The most frequent themes related to the goal of the paper were “(a) racialization of the virus as a multi-faceted threat; (b) COVID-19 disease as a threat; (c) calls for collectivization to curb the racialization of the virus; and (d) offers of speculative solutions to end discrimination.” (p. 9). [29] examine the transgenerational divide in the portrayal of children, young adults, adults and the elderly in pictures published in newspapers articles about the COVID-19 pandemic. They find that children were often presented as controlled or playful, young adults as students or party-goers, adults as responsible people (e.g., professionals, experts, caretakers) and the elderly as isolated. The composition of the pictures was also found to be different depending on the group (e.g., elderly often photographed alone behind windows or on their balcony, with a large framing; children photographed in groups in the 'playful' representation, or alone with a tight framing in the 'controlled' representation).

In sum, the few examples of studies provided above show that the qualitative analysis of press articles has been fruitful in the understanding of sensemaking in relation to emerging infection diseases, but such enquiry benefits from more automatized methods (natural language processing) when dealing with thousand articles or more, for connected reasons: a) it is free from human biases; b) allows to process large amounts of documents rapidly; and 3) there is no need for human coders (feasibility increased).

## Topic models

Topic modelling is a generative probabilistic clustering algorithm that groups words into topics (operationalizations of themes), or coherent sets of words that cluster together across a corpus of text [30]. Topic modelling allows identifying co-occurring word patterns and extracting the underlying topic distribution for each text document in a corpus. In topic modelling, instead of coding topics by hand, like in the previously mentioned studies, topics are generated from the data in an emergent fashion, rather than from the words that researchers believe theoretically represent that category.

Several studies have relied on topic models for the discovery of emergent topics in textual productions related to emerging infectious diseases. For instance [31], examined the discussions on Twitter relating to the Zika outbreak of 2015 (about 4 million Tweets). They performed a selection of relevant topics from 50 topics extracted. Examples of the topics they found are: conspiracy theories, environmental concerns, negative effects, viral testing, vaccination, and US politics. With regards to the COVID-19 pandemic [32], examined social media discussions on a diversity of platforms (Twitter, Instagram, YouTube, Reddit and Gab) in order to compare the topics of discussion and user engagement between platforms as well as the quality of the sources cited. They found some differences between platforms in terms of engagement notably. The topic “death toll & infection rates” was among the top three topics on four of the five considered platforms. Top topics (e.g., biological warfare, government and decision making, economic impact, protection advice) were otherwise heterogeneous between platforms. [33] performed topic modelling (setting the number of topics to 15) on about 7000 web articles and 17,000 comments on COVID-19 published between January and September 2020. Here are examples of the topics they found, some of which might be specific to their data: Earthquake during pandemics, Elections in Croatia, Crime, Online education, Anti-pandemic measures protest, Pandemic worldwide, Pandemic in Croatia, Economy.

In sum, the examples of research presented above have shown the potential of topic modelling for the automatic extraction of topics from texts related to infectious diseases.

## The present study

The Omicron variant appeared on the world stage when governments of different nations (e.g., the United States and the United Kingdom) had prepared the population to the end of the pandemic (the endemic stage). Two rather opposed strategies were in place in these regions: A vaccination mandate strategy in the United States [34], and the end of almost all restrictions in the United Kingdom [35]. Therefore, in the United Kingdom particularly, the emergence of Omicron has caused an important shift in the official policy (e.g., Plan B, enacted on December 8 2021 [36]) and a shock to the population who had been living under normal circumstances for a few months and was prepared to seeing a rapid end to the pandemic. Under such circumstances particularly, the need for sensemaking might be paramount [37] and the search for information in press articles provides a way to reduce uncertainty.

How an issue is discussed in press articles provides basis for sensemaking and changes in associated topics reflects the evolving nature of how the issue is understood [21]. In the present study, we investigate the evolution of themes emerging from UK press articles on the Omicron variant of SARS-Cov2 published between November 26 and December 31st 2021. This period thus includes the emergence of the Omicron variant in South Africa and the moment it became the dominant variant in all regions of the United Kingdom [38]. This notably allows for the examination of the changes in the representation of themes as the variant became a more and more concrete risk in the UK. We provide this analysis at different levels of granularity, testing differences between weeks and linear trends within weeks. We also comment on the general pattern over the considered period. Finally, we will discuss our finding from the perspective of sensemaking and the management of risk.

## Method

### Material

Data was collected in January (the 9th, 2022), via Lexis-Nexis (an online portal that allows to collect newspaper print and online articles upon subscription, see [21] for a similar work). We sought to retrieved documents published in a selection of British Newspapers between the 24^th^ of November 2021 (the day of the communication of the discovery of the Omicron variant) and the 31^st^ of December 2021. We could not retrieve newspaper articles published in the first two days. The dates of publication of the obtained articles therefore range from November 26th to December 31st 2021. The list of Newspapers (see Table 1) includes the broadsheet and tabloid newspapers indicated on the Wikipedia page listing the newspapers in the United Kingdom.^1^ As inclusion criterion, we searched for articles whose title included the word “Omicron” and further restricted our results to English language articles.

**Table 1:** Documents count by newspaper

Newspaper	N docs
The Independent (United Kingdom)	654
The Guardian (London)	210
The Daily Telegraph (London)	110
The Times (London)	86
Daily Mirror	79
The Sun (England)	71
The Independent—Daily Edition	47
Financial Times (London, England)	32
Daily Mail (London)	28
The Sunday Times (London)	26
The Sunday Telegraph (London)	25
City A.M	23
Metro (UK)	18
The Evening Standard (London)	12
The Observer (London)	12
Sunday Express	6
Daily Star	4
Mail on Sunday (London)	4
Daily Star Sunday	1

*Document cleaning.* From Lexis-Nexis, we retrieved 2,948 documents. We imported text documents in R [39]. In order to remove duplicated documents, besides removing documents whose text was an exact replica of other documents, we created a document similarity matrix relying on the Jaccard distance using the package *textreuse* [40]. We visually inspected documents at different threshold of similarity (that ranges from 0 to 1, i.e., no word overlaps and perfect match, respectively) and removed documents of which the similarity was above 0.70. These documents were highly similar, with some negligible differences. This procedure left us with 1,478 documents. Finally, we removed documents whose length was above 2.5 standard deviations (SD) from the mean of our corpus wordcount (max length was 40,127 words). Our final corpus was composed of 1,448 documents, the length of which is 688 words per document (SD = 517, range: 9 – 8,500). Documents (articles) dates span from 26^th^ November to 31^st^ December (median 12^th^ December).

### Measures

*Text preprocessing.* Text preprocessing for topic extraction relied on the corpus’ document-term matrix (DTM), namely a bag of words for the whole corpus that stores the occurrences of each word (columns) for each document (rows). To generate the DTM, we relied on the *quanteda* R package [41]. The pipeline was as follow: Remove non-ASCII characters; convert upper case to lower case; remove URLs, remove punctuation, remove numbers, remove separators, split hyphens, and remove symbols; remove stopwords (175 English stopwords from the *stopwords* R package [42]; word stemming (words were reduced to their root, e.g., “frequenc” for “frequency” and “frequencies”); Tokenization (splitting the text into separate terms); and finally generate the DTM, which was finally composed of 16,519 unique words, for a total of 564,483 non-unique words (98.58% sparse). The data from this step (DTM) are available on osf.io: https://osf.io/ft9vm.

*Topic extraction.* Topics were extracted with Latent Dirichlet Allocation, (LDA [30]). By setting a priori a value for number of topics desired, LDA computes, for each document in the corpus, the probabilities for all topics to be represented in the document: the machine scans word co-occurrences within documents based on the properties of the corpus and returns a matrix with word-in-topic and topic-in-document probabilities. In other words, a word *w* has probability *β* of being part of topic *k*; a topic *k* has probability *γ* of being part of document *n*. The sum of all the word probabilities within one topic is 1, and the sum of all the topic probabilities within one document is 1.

Although unsupervised, LDA topic modelling requires the researchers to set a number of topics (*k*) desired. In the exploratory part, in order to assess a mathematical solution to the number of topics for our corpus, we started in an unsupervised fashion, relying on the R package *ldatuning* [43] that provides a function to estimate the number of topics within a corpus based on four different metrics obtained via an unsupervised fashion. We set the *ldatuning* function to explore the fits of topics for a vector of *k*s = {5, 10, 15, 20, 25, 30, 35, 40, 45, 50}. The function returned k = 50 as the number of topics which optimized the values computed by most algorithms. Topic extraction was performed with the *topicmodels* R package [44], using Gibbs sampling. We left the other LDA parameters set as default, while setting the same seed for reproducibility for all topic extractions.

For the purpose of validation, we examined the cosine similarity of the LDA documents gamma weights at the document level with the document level codings of ChatGPT. For each topic, we selected 2 articles in the highest tertile in terms of gamma values. Documents that were selected for several topics were replaced so that we obtained 100 documents in total for the validation step. We completed the following instructions with the content of each of these article (placed between <  <  > >).

Here is a newspaper article: << ARTICLE_CONTENT_HERE >> I want you to code whether the 50 topics below, composed of sets of words, represent the content of the article, on an integer scale of 0 to 10, 10 meaning they represent the content perfectly. Consider the meaning of the words in the topics, and the extent to which the meaning is present in the articles, rather than count the words. Here are the 50 topics for you to grade on this article [Topic 1: "variant africa south case omicron said new health countri first", Topic 2: "peopl new restrict year close limit event day rule also", Topic 3: "virus mutat antibodi covid treatment immun drug protein spike use", Topic 4: "booster vaccin jab said get peopl dose omicron nhs protect", Topic 5: "minist johnson govern restrict said prime plan new bori rule", Topic 6: "market stock share price trade investor ftse point drop fell", Topic 7: "one now can get even time just know make like", Topic 8: "omicron new covid variant plan christma hour part look six", Topic 9: "case omicron covid number day peopl infect week uk report", Topic 10: "lockdown high public restrict sinc covid pandem remain plan professor", Topic 11: "rate bank economi econom rise inflat omicron uk expect month", Topic 12: "school children educ said close govern term teacher home keep", Topic 13: "travel test uk arriv countri day restrict rule take isol", Topic 14: "parti polit elect year right lockdown independ tori now vote", Topic 15: "year london retail shop said sale citi mani level week", Topic 16: "variant omicron transmiss case vaccin spread said concern delta alreadi", Topic 17: "per cent yesterday year averag last day daili just anoth", Topic 18: "time publish block gmt say govern year decemb updat today", Topic 19: "test day posit later flow isol pcr result contact take", Topic 20: "scotland case said sturgeon first scottish minist govern ms omicron", Topic 21: "vaccin omicron dose protect variant pfizer booster two said effect", Topic 22: "omicron variant covid new mean press bori give look news", Topic 23: "came santa park found look small season far date thought", Topic 24: "us new vaccin state said biden presid get york covid", Topic 25: "christma peopl parti cancel year go famili event festiv keep", Topic 26: "countri said franc european europ germani minist vaccin restrict govern", Topic 27: "nhs hospit staff servic patient said covid care london health", Topic 28: "letter year say world first die stori talk name compani", Topic 29: "year pandem said holiday book summer restrict next pre demand", Topic 30: "said go think told ad uk prof peopl bbc come", Topic 31: "vaccin countri world pandem global access long need must nation", Topic 32: "said case peopl ireland health govern new dr come chief", Topic 33: "claim public person allow post rather messag social email detail", Topic 34: "javid health secretari mr sajid yesterday page variant ask england", Topic 35: "model scenario measur death peak scientist januari may govern next", Topic 36: "busi support said govern sector restaur industri hospit trade pub", Topic 37: "game club player last footbal match show leagu posit sport", Topic 38: "countri india china japan new case report first control citi", Topic 39: "mask wear face peopl public cover shop requir transport use", Topic 40: "year group expect million releas oil deal opec one firm", Topic 41: "omicron infect sever delta less data hospit peopl hospitalis wave", Topic 42: "coronavirus said omicron new surg u. health pandem covid infect", Topic 43: "flight cancel airlin travel train due fli passeng disrupt run", Topic 44: "northern ireland covid health public case minist need first monday", Topic 45: "work home offic plan return staff worker move back continu", Topic 46: "covid symptom dr mild peopl patient new cold doctor medic", Topic 47: "roll time uk coronavirus one meet christma put govern econom", Topic 48: "govern measur sage need group advis uk scientif said time", Topic 49: "australia state covid case report south australian time wale new", Topic 50: "week last start sinc month one year pandem recent point"]. Again, rate each of the topics.

The responses were parsed to obtain a data frame containing 100 rows (corresponding to the randomly selected documents) and 50 columns (corresponding to the coding of each topic). We computed the cosine similarity of the gamma values of each document with the ChatGPT codings. The corresponding script and data are in the OSF repository mentioned above. We obtained an appropriate to good correspondence of the LDA gamma weights with the ChatGPT-coded topic representation as cosine similarity values ranged from 0.30 to 0.80, with median and average values of 0.53.

### Topic interpretation

**Fig. 1:**
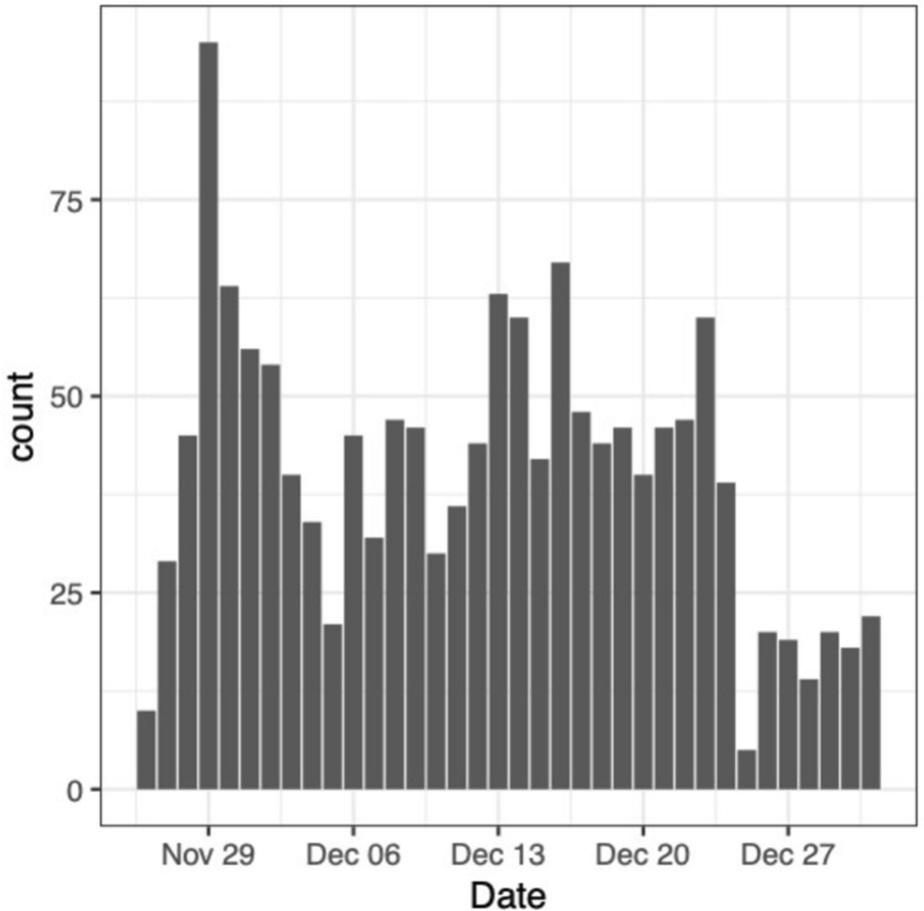
Document count distribution by day

We provide the top 15 words for each topic (See Fig. 1) which taken together summarize the topic’s content [45]. In the text, we also provide a general interpretation of the topics relying mainly upon the first 5 top words (highest beta values). We also consulted the press articles in which the topic is most represented if necessary (the articles with the highest gamma values).

### Data visualisation and analysis

In order to explore each topic trend over time, we averaged the topic’s gamma values, namely the probability a topic is part of a document (i.e., articles), for each day and created a time series of gamma values over days. Such a method has been shown capable of reliably associating LDA topics extracted from documents with real-world events such as outbreak of diseases (e.g., cholera, influenza, Zika virus, covid-19), death of societally significant people (e.g., Osama bin Laden, Michael Jackson) and wars [46, 47]. We created a time series for each topic. We then analyzed change in the probability of each topic to be represented in documents by week. In creating weekly-based bins, we are capable of detecting evolving trends in themes. A similar procedure was previously used to extract common structures underlying narratives, dividing texts into *n* segments and extracting a common narrative arc [48]. We therefore assigned relevant documents to one of four weeks (from Monday to Sunday): Week 1: from 29^th^ November to 5^th^ December (N documents = 364); Week 2: from 6^th^ December to 12^th^ December (N = 280); Week 3: from 13^th^ December to 19^th^ December (N = 370); and Week 4: from 20^th^ December to 26^th^ December (N = 257). We decided to divide our time frame into four weeks because document number in each week was balanced (hence we excluded from this analysis N = 84 documents between 26^th^-28^th^ November and N = 93 documents between 27^th^ – 31^st^ December).

For the purpose of assessing the evolution of each topic within our time frame, we performed an Analysis of Variance (ANOVA) on the gamma values of each week. We also performed a post hoc Tukey’s HSD (honestly significant difference) test for all possible pairwise week comparisons in order to test in which week the topic was represented the most/least. Furthermore, for each topic, we also provide a measure of within-week slope so to offer an estimate of the linear evolution of topic within the week under consideration. This was achieved by running a regression of gamma values over the seven days of the week. We also present the standardized coefficient (*β*) along with standard errors (SE) and *p* values (Table 2).

**Table 2:** Statistical tests in relation to the considered topics: omnibus ANOVA and regression of time on gamma (probability of documents to pertain to each topic) for each considered week

Title	Top words	F (3,1267)	Sig	w1	w2	w3	w4
A1. Origins Omicron	"variant africa south case omicron said new health countri first"	54.94	***	-0.06 (0.03) *	-0.1 (0.03) ***	-0.01 (0.03)	-0.01 (0.04)
A2. Virus mutations	"virus mutat antibodi covid treatment immun drug protein spike use"	5.03	**	0.04 (0.03)	-0.02 (0.03)	0.01 (0.03)	-0.02 (0.04)
A3. News of new variant of Covid	"omicron variant covid new mean press bori give look news"	56.90	***	0.01 (0.03)	-0.07 (0.03) *	-0.05 (0.03)	-0.06 (0.04)
B1. Vaccination, transmission and cases	"variant omicron transmiss case vaccin spread said concern delta alreadi"	21.54	***	0.02 (0.03)	-0.05 (0.03)	-0.03 (0.03)	-0.09 (0.04) *
B2. Booster vaccination program	"booster vaccin jab said get peopl dose omicron nhs protect"	11.16	***	-0.07 (0.03) *	0.16 (0.03) ***	-0.22 (0.02) ***	0 (0.04)
B3. Reports of record rises in infections	"case omicron covid number day peopl infect week uk report"	12.37	***	0.07 (0.03) **	0.02 (0.03)	0.03 (0.03)	0.13 (0.04) ***
B4. Protection of two vaccine doses with booster	"vaccin omicron dose protect variant pfizer booster two said effect"	7.30	***	-0.02 (0.03)	0.01 (0.03)	-0.06 (0.03) *	-0.05 (0.04)
B5. Hospitalisations and severity	"omicron infect sever delta less data hospit peopl hospitalis wave"	23.15	***	0.06 (0.03) *	0.08 (0.03) **	0.02 (0.03)	0.1 (0.04) **
B6. The pandemic, infections and health during the holidays	"coronavirus said omicron new surg u. health pandem covid infect"	3.03	*	0.04 (0.03)	-0.05 (0.03)	0 (0.03)	-0.07 (0.04)
B7. Covid symptoms, patients and medical care	"covid symptom dr mild peopl patient new cold doctor medic"	2.46		-0.04 (0.03)	0 (0.03)	-0.03 (0.03)	0.07 (0.04)
B8. Vaccination around the world	"vaccin countri world pandem global access long need must nation"	2.10		-0.03 (0.03)	-0.01 (0.03)	-0.02 (0.03)	-0.05 (0.04)
C1. International travel, testing and isolation	"travel test uk arriv countri day restrict rule take isol"	24.57	***	-0.08 (0.03) **	-0.07 (0.03) *	-0.01 (0.03)	-0.07 (0.04) *
C2. Mask-wearing in transportation and stores	"mask wear face peopl public cover shop requir transport use"	13.04	***	-0.08 (0.03) **	0 (0.03)	-0.04 (0.03)	-0.08 (0.04) *
C3. Scenarios, measures, peaks and death	"model scenario measur death peak scientist januari may govern next"	16.96	***	0.05 (0.03)	0.12 (0.03) ***	0.01 (0.03)	0.03 (0.04)
C4. Seasons, holidays and restrictions	"year pandem said holiday book summer restrict next pre demand"	5.13	**	0.01 (0.03)	-0.05 (0.03)	0.05 (0.03)	0.01 (0.04)
C5. Financial help in different sectors	"busi support said govern sector restaur industri hospit trade pub"	9.44	***	0.05 (0.03)	0.03 (0.03)	0.09 (0.03) ***	-0.06 (0.04)
C6. Christmas and the new variant	"omicron new covid variant plan christma hour part look six"	58.15	***	-0.05 (0.03)	0.03 (0.03)	0.02 (0.03)	-0.12 (0.04) ***
C7. Restrictions and New Year celebrations	"peopl new restrict year close limit event day rule also"	19.83	***	0.03 (0.03)	0.02 (0.03)	0.05 (0.03)	0.01 (0.04)
C8. Lockdown and public restrictions	"lockdown high public restrict sinc covid pandem remain plan professor"	1.66		0 (0.03)	-0.01 (0.03)	0.01 (0.03)	-0.04 (0.04)
C9. Close schools, keep children home	"school children educ said close govern term teacher home keep"	1.59		-0.04 (0.03)	-0.02 (0.03)	-0.07 (0.03) **	-0.03 (0.04)
C10. PCR tests positive results and isolation	"test day posit later flow isol pcr result contact take"	1.70		0.01 (0.03)	0.01 (0.03)	-0.07 (0.03) **	0.01 (0.04)
C11. Home office and work	"work home offic plan return staff worker move back continu"	2.22		0.03 (0.03)	0.01 (0.03)	0.01 (0.03)	0.02 (0.04)
D1. Prime minister statements	"minist johnson govern restrict said prime plan new bori rule"	7.73	***	-0.02 (0.03)	0.04 (0.03)	-0.08 (0.03) **	-0.04 (0.04)
D2. Health secretary statements	"javid health secretari mr sajid yesterday page variant ask england"	3.20	*	-0.06 (0.03) *	-0.09 (0.03) **	-0.1 (0.03) ***	0.03 (0.04)
D3. Government's SAGE advisers	"govern measur sage need group advis uk scientif said time"	3.97	**	0.05 (0.03)	0.07 (0.03) *	0.12 (0.03) ***	-0.03 (0.04)
D4. First minister (Scotland) statements	"scotland case said sturgeon first scottish minist govern ms omicron"	10.50	***	-0.04 (0.03)	-0.04 (0.03)	0.01 (0.03)	0 (0.04)
D5. Health communications of the Irish government	"said case peopl ireland health govern new dr come chief"	3.00	*	0.06 (0.03) *	0.01 (0.03)	0.07 (0.03) **	-0.04 (0.04)
D6. Northern Ireland health communications	"northern ireland covid health public case minist need first monday"	2.37		-0.02 (0.03)	0.01 (0.03)	-0.06 (0.03) *	0.07 (0.04)
D7. Public declarations, social media and e-mail	"claim public person allow post rather messag social email detail"	0.49		0.01 (0.03)	0.02 (0.03)	-0.03 (0.03)	0.05 (0.04)
E1. Situation in the US	"us new vaccin state said biden presid get york covid"	6.30	***	-0.02 (0.03)	-0.06 (0.03) *	0.09 (0.03) ***	-0.07 (0.04) *
E2. Situation in European countries	"countri said franc european europ germani minist vaccin restrict govern"	5.79	***	-0.07 (0.03) **	-0.06 (0.03) *	0.08 (0.03) **	-0.1 (0.04) **
E3. Situation in Asia	"countri india china japan new case report first control citi"	3.84	**	-0.01 (0.03)	-0.04 (0.03)	-0.02 (0.03)	0.06 (0.04)
E4. Situation in Australia	"australia state covid case report south australian time wale new"	1.42		-0.01 (0.03)	0.02 (0.03)	-0.01 (0.03)	0.02 (0.04)
F1. Market and investing	"market stock share price trade investor ftse point drop fell"	4.36	**	0 (0.03)	-0.09 (0.03) **	-0.03 (0.03)	0 (0.04)
F2. Politics	"parti polit elect year right lockdown independ tori now vote"	2.27		-0.03 (0.03)	0.02 (0.03)	-0.01 (0.03)	0.07 (0.04) *
F3. Concerns over the NHS staff and patient care	"nhs hospit staff servic patient said covid care london health"	18.27	***	0 (0.03)	0.09 (0.03) **	0.04 (0.03)	0 (0.04)
F4. Retail sales	"year london retail shop said sale citi mani level week"	11.95	***	0.05 (0.03) *	-0.01 (0.03)	0.09 (0.03) ***	0.13 (0.04) ***
F5. Airlines and flight cancelations	"flight cancel airlin travel train due fli passeng disrupt run"	5.72	***	0.04 (0.03)	-0.03 (0.03)	0.01 (0.03)	0.08 (0.04) *
F6. Economy and inflation	"rate bank economi econom rise inflat omicron uk expect month"	2.70	*	0.07 (0.03) *	0.03 (0.03)	0 (0.03)	0.02 (0.04)
F7. Christmas party cancellations	"christma peopl parti cancel year go famili event festiv keep"	2.03		0.11 (0.03) ***	-0.01 (0.03)	0.03 (0.03)	-0.04 (0.04)
F8. Sports	"game club player last footbal match show leagu posit sport"	2.11		-0.04 (0.03)	0.01 (0.03)	0.02 (0.03)	-0.02 (0.04)
F9. Christmas, the coronavirus and the economy	"roll time uk coronavirus one meet christma put govern econom"	1.38		0.04 (0.03)	-0.07 (0.03) *	0.02 (0.03)	-0.08 (0.04) *
G1. ??	"one now can get even time just know make like"	2.85	*	0.01 (0.03)	0.01 (0.03)	0.04 (0.03)	-0.06 (0.04)
G2. ??	"time publish block gmt say govern year decemb updat today"	0.65		0.01 (0.03)	-0.06 (0.03) *	-0.04 (0.03)	0.06 (0.04)
G3. ??	"came santa park found look small season far date thought"	0.17		0.01 (0.03)	0.02 (0.03)	0.06 (0.03) *	-0.03 (0.04)
G4. ??	"letter year say world first die stori talk name compani"	0.69		-0.02 (0.03)	-0.01 (0.03)	0.01 (0.03)	0 (0.04)
G5. ??	"said go think told ad uk prof peopl bbc come"	7.74	***	0.04 (0.03)	-0.14 (0.03) ***	-0.01 (0.03)	-0.07 (0.04)
G6. ??	"year group expect million releas oil deal opec one firm"	0.95		0.04 (0.03)	0.01 (0.03)	0.02 (0.03)	-0.05 (0.04)
G7. Values and durations	"per cent yesterday year averag last day daili just anoth"	8.03	***	0.03 (0.03)	0 (0.03)	0.03 (0.03)	0.1 (0.04) **
G8. Durations	"week last start sinc month one year pandem recent point"	2.91	*	0.06 (0.03) *	-0.02 (0.03)	0.07 (0.03) **	0.01 (0.04)

Note: *p* < .1, *: *p* < .05, **: *p* < .01, ***: *p* < .001

## Results

The distribution of the documents by date is depicted in Fig. 1. One can see that the first day of press coverage featured less articles than every other day, except for December 25 (Christmas). From that point, the press coverage increased to reach its maximum on November 29 and decrease again. December 5 and the period from Christmas until the end of the year featured a lesser coverage of the variant. In Table 1, we report document count by newspaper. The Independent, The Guardian, The Daily Telegraph and The Times were the newspapers with the most articles about Omicron, with more than 100 articles published in each. Less than 10 articles were published in each of the following outlets: Sunday Express, Daily Star, Mail on Sunday, Daily Star Sunday. We note three of these are published once a week only.

### Topic group A: A new variant

**Fig. 2:**
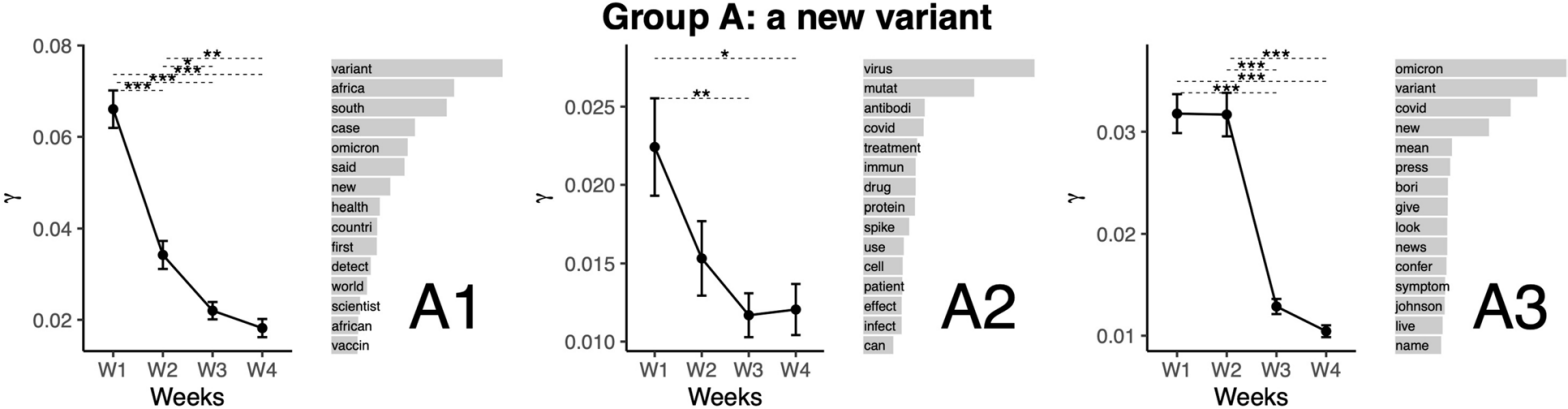
Top words (beta values), mean comparisons of representation (gamma values) for topics in Group A: A new variant. Error bars represent the standard error of the mean. P values are obtained from the Tukey HSD pairwise comparison on the ANOVA

We grouped the topics for the presentation of the results. We titled the first group of topics "A new variant" (see Fig. 2). The first presented topic (A1) is related to the Origin of Omicron in South Africa. This topic featured the highest mean gamma values (which we will refer to as the representation of the topics from here) at Week 1. The representation of the topic was lower for all other weeks). This topic was also less represented during Weeks 3 and 4 compared with Week 2. Topic A2 relates to the mutations present in the Omicron variant. This topic featured the highest representation at Week 1. The representation of the topic was lower at Weeks 3 and 4. Topic A3 relates to the mention of news regarding Omicron, a new variant of SARS-CoV2. It representation was highest at Weeks 1 and 2, from which other weeks differed.

### Topic group B: Symptoms, cases and vaccines

**Fig. 3:**
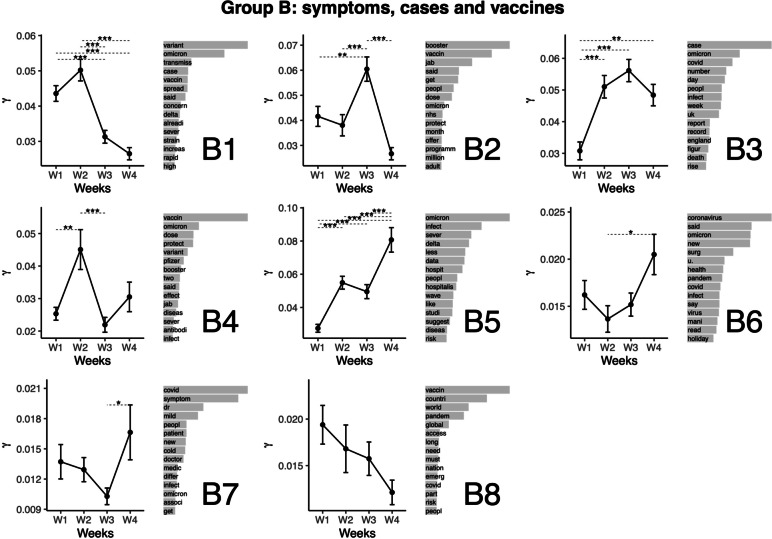
Top words (beta values), mean comparisons of representation (gamma values) for topics in Group B: Symptoms, cases and vaccines. Error bars represent the standard error of the mean. P values are obtained from the Tukey HSD pairwise comparison on the ANOVA

We titled the second group of topics "Symptoms, cases and vaccines" (see Fig. 3). In that group, Topic B1 relates to transmissions, cases and vaccination. This topic featured the highest representation at Weeks 1 and 2, from which the representation was lower at Weeks 3 and 4. Topic B2 relates to the booster vaccination program. This topic featured the highest representation at Week 3, corresponding with the availability of booster vaccine doses to all eligible adults [49]. The representation of the topic was lower for all other weeks. Topic B3 relates to reports of record rises in infections. This topic featured the lowest representation at Week 1. The representation was higher for other weeks. Topic B4 relates to protection of two vaccine doses with booster. This topic featured the highest representation at Week 2, from which Weeks 1 and 3 (lower representation) differed. Topic B5 relates to hospitalisations, infections and severity. This topic featured the highest representation at Weeks 4. The representation was lower for other weeks. Additionally, the representation of the topic at Week 1 was lower than at Weeks 2 and 3, which converges with the increase in infections and hospitalizations. Topic B6 relates to the pandemic, infections and health during the holidays. This topic featured the highest representation at Weeks 4, from which the representation was lower at Week 2. Other weeks had a mean closer to that of Week 2 than Week 4, but the mean differences to Week 4 did no reach significance. Topic B7 relates to Mild symptoms, patients and medical care. This topic featured the highest representation at Week 4, from which Week 3 (lower value) differed. Topic B8 relates to vaccination around the world. A non-significant declining trend is observable on the graph of the representation though time.

### Topic group C. Restrictions and measures (excl. vaccination)

**Fig. 4:**
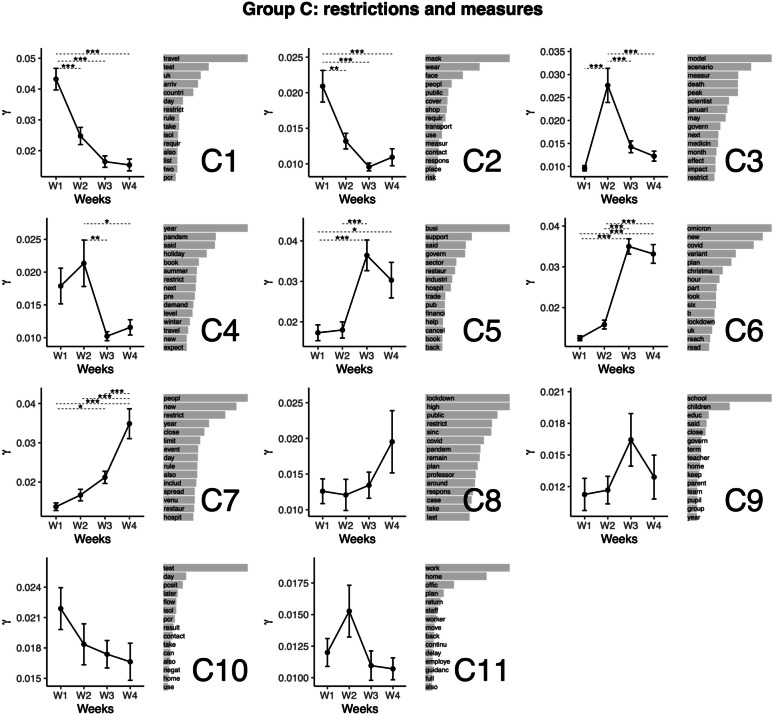
Top words (beta values), mean comparisons of representation (gamma values) for topics in Group C: Restrictions and measures (excl. vaccination). Error bars represent the standard error of the mean. *P* values are obtained from the Tukey HSD pairwise comparison on the ANOVA

The next group of topics relates to restrictions and measures (see Fig. 4). Topic C1 relates to international travel, testing and isolation. This topic featured the highest representation at Week 1. The representation was lower for other weeks. This pattern corresponds with the initial ban on international travel (and related measures), which is of common knowledge [50]. Topic C2 relates to mask-wearing in transportation and stores (mandated in Great Britain on November 27, 2021 [51]). This topic featured the highest representation at Week 1. The representation was lower for other weeks. Topic C3 relates to the modeling of scenarios, the establishment of measures, and the estimations of peaks and deaths. This topic featured the highest representation at Week 2. The representation was lower for other weeks. Topic C4 relates to periods (year; seasons – summer, winter; holidays) and restrictions. This topic featured the highest representation at Week 2, from which Weeks 3 and 4 (lower values) differed. Topic C5 relates to financial help to businesses in different sectors. This topic featured the highest representation at Weeks 3. Weeks 1 had a lower representation of the topic compared with Weeks 3 and 4. On December 21, the Department for Business, Energy & Industrial Strategy (2021, Dec 21) announced a 1 billion budget to support impacted businesses [52]. Topic C6 relates to Christmas, the new variant and Plan B. This topic featured the highest representation at Weeks 3 and 4, compared to which the representation was lower at Weeks 1 and 2. Topic C7 relates to restrictions and the New Year. This topic featured the highest representation at Week 4. The representation of the topic was lower for all other weeks. This topic was also more represented during Week 3 compared with Week 1. Again, the pattern of representation of this topic corresponds with the period (New Year) featured in the top terms. The four next topics didn’t feature significant variation between the weeks: Topic C8 relates to lockdown and public restrictions. Topic C9 relates to closing schools and keeping children home. Topic C10 relates to PCR tests positive results and isolation. Topic C11 relates to home office and work.

### Topic group D: Advice and communication (UK)

**Fig. 5:**
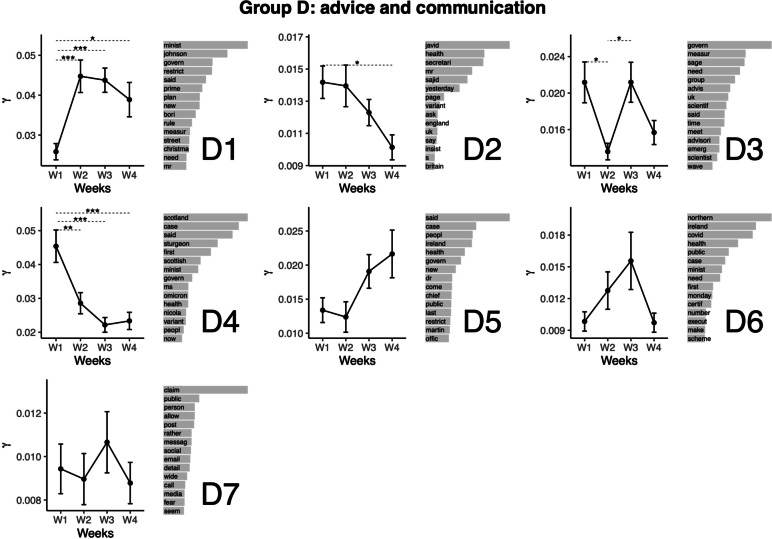
Top words (beta values), mean comparisons of representation (gamma values) for topics in Group D: Advice and communication. Error bars represent the standard error of the mean. P values are obtained from the Tukey HSD pairwise comparison on the ANOVA

The next group of topics relates to advice and communication (see Fig. 5). Topic D1 relates to the Prime Minister’s statements on restrictions, notably Plan B. This topic featured the lowest representation at Week 1, for which the representation was higher during other weeks. Topic D2 relates to Health Secretary statements. This topic featured the highest representation at Weeks 1 and 2. The representation of this topic was lower at Week 4 compared with Week 1. Topic D3 relates to the Government’s Scientific Advisory Group for Emergencies (SAGE) recommendations. This topic featured the highest representation at Weeks 1 and 3, from which Week 2 (lower value) differed. Topic D4 relates to statements of the First Minister of Scotland. This topic featured the highest representation at Week 1. The representation was lower for other weeks. Topic D5 relates to health communications of the Irish government. This topic didn’t feature significant variation across the weeks. Topic D6 relates to health communications regarding Northern Ireland. This topic didn’t feature significant variation across the weeks. Topic D7 relates to public declarations, mentions of social media and e-mail. This topic didn’t feature significant variation across the weeks.

### Topic group E. Situation in foreign countries and regions

**Fig. 6:**
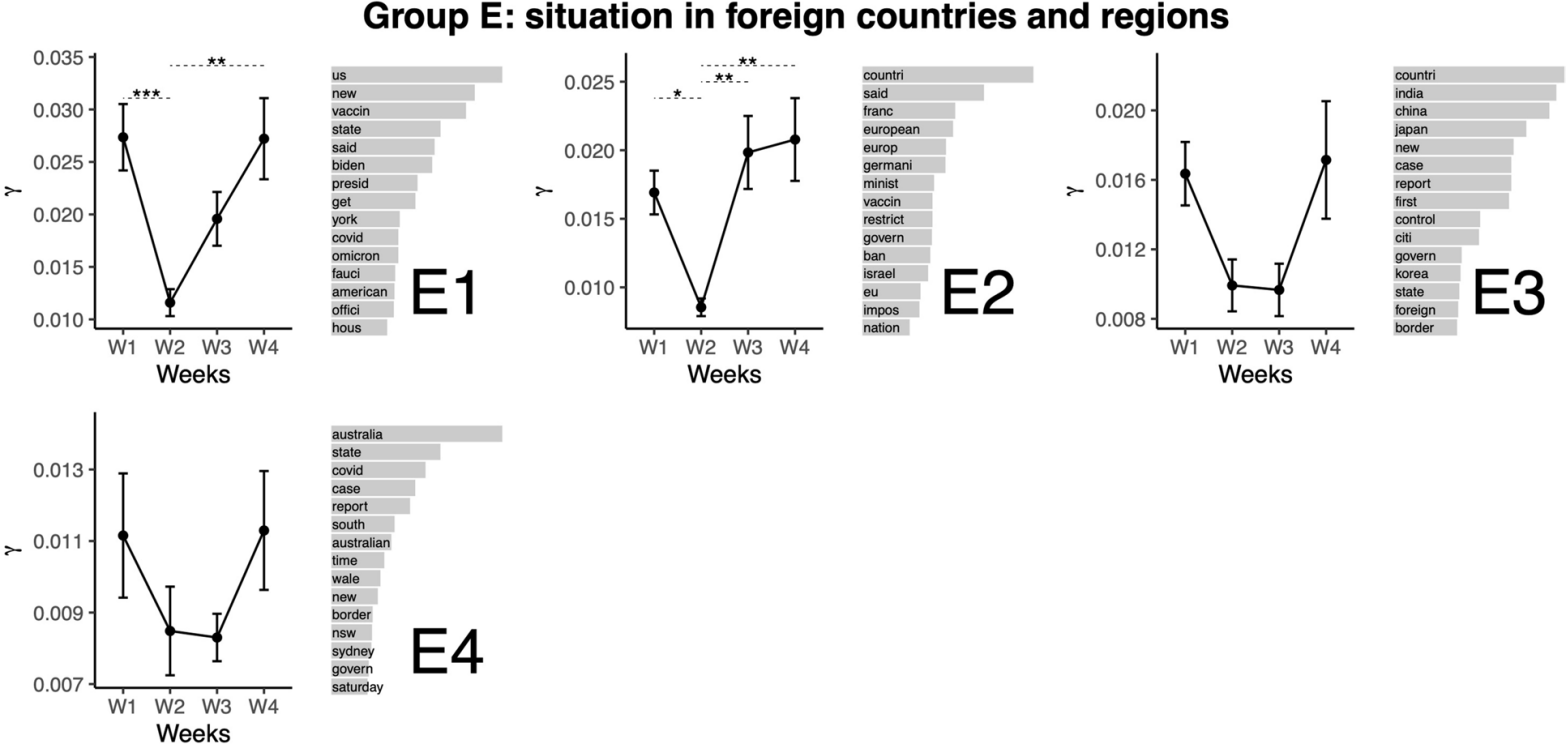
Top words (beta values), mean comparisons of representation (gamma values) for topics in Group E: Situation in foreign countries and regions. Error bars represent the standard error of the mean. P values are obtained from the Tukey HSD pairwise comparison on the ANOVA

The next group of topics relates to the situation in foreign countries and regions, and corresponding statements (see Fig. 6). Topic E1 relates to situation in the US. This topic featured the lowest representation at Week 2, from which Weeks 1 and 4 (higher values) differed. Topic E2 relates to situation in European countries. This topic also featured the lowest representation at Week 2. The representation was higher for other weeks. Topics E3 and E4 relate to the situation in Asia and Australia, respectively. Neither of these topics featured significant variation across the weeks, although visually the curves indicate a similar pattern as for topic E1.

### Topic group F: Domains and events

**Fig. 7:**
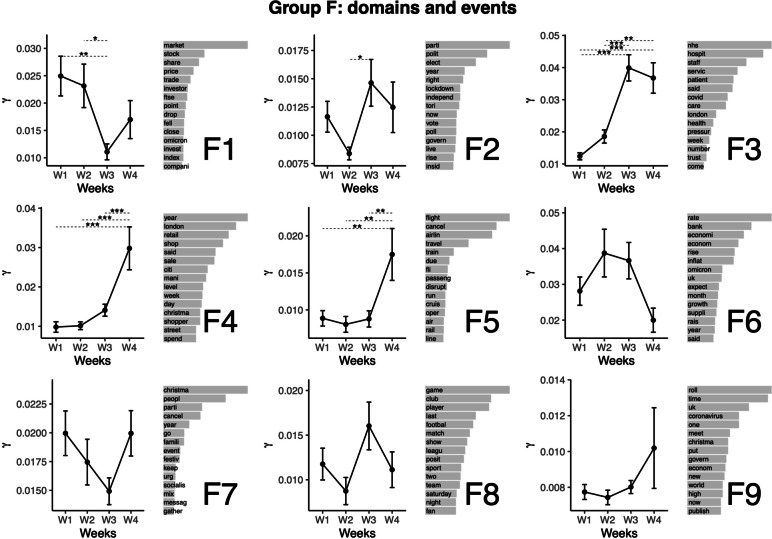
Top words (beta values), mean comparisons of representation (gamma values) for topics in Group F: Domains and events. Error bars represent the standard error of the mean. P values are obtained from the Tukey HSD pairwise comparison on the ANOVA

We titled the last group of topics “Domains and events” (e.g., sports, economy, Christmas) (see Fig. 7). Topics classified in this group relate to domains and events that might be affected by the pandemic. In this group, Topic F1 relates to market and investing. This topic featured the highest representation at Weeks 1 and 2, from which the representation was lower at Week 3. Topic F2 relates to politics. This topic featured the highest representation at Week 3, from which only Week 2 (lower value) differed. Topic F3 relates to the NHS staff and patient care. This topic featured the highest representation at Weeks 3 and 4. The representation was lower for the other weeks. Topic F4 relates to retail sales. This topic featured the highest representation at Week 4. The representation was lower for other weeks. Topic F5 relates to Airlines and flight cancelations. This topic featured the highest representation at Week 4. The representation was lower for other weeks. The following topics didn’t feature significant variation between the weeks: Topic F6 (economy and inflation), F7 (Christmas party cancellations), F8 (sports), F9 (Christmas, the coronavirus and the economy).

### Topic group G: Other topics

**Fig. 8:**
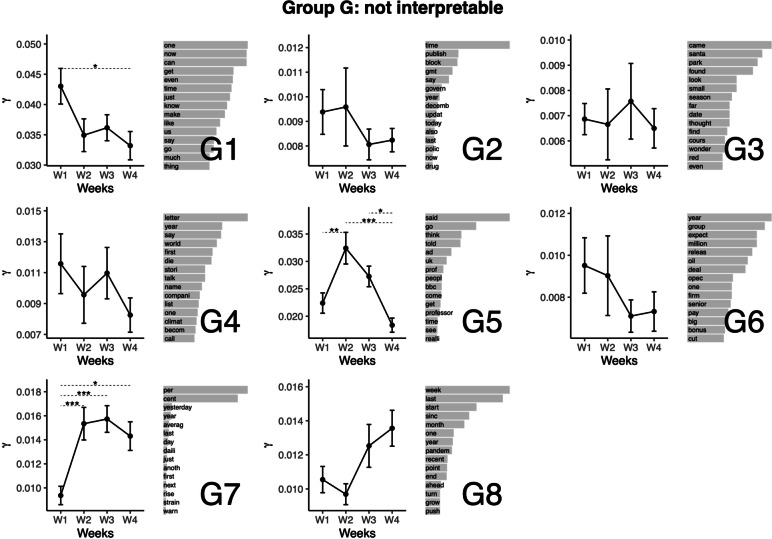
Top words (beta values), mean comparisons of representation (gamma values) for topics in Group G: Other topics. Error bars represent the standard error of the mean. P values are obtained from the Tukey HSD pairwise comparison on the ANOVA

Other topics (see Fig. 8). We placed in this group topics which were not much interpretable (Topics G1 to G6), or focused almost exclusively on durations (Topics G7, G8). We do not comment on these topics further.

### Summary of the results

All three topics (A1 to A3) classified in the group "A new variant" featured higher representation in the early days of the emergence of Omicron. Of the topics in the group “Symptoms, cases and vaccines”, two topics featured a higher representation at Week 2 (B1. Transmission, cases and vaccination B4. Booster and double jab protection) and Week 3 (B3. Rise in infections, B2. Booster vaccination program), and three at Week 4 (B5. Hospitalisation, B6. Pandemic and holidays, B7. Mild symptoms and care), while Topic B8 (vaccination around the world) didn’t vary significantly. Topics in the group “Restrictions and measures (excl. vaccination)” featured an irregular pattern, with highest values at Week 1 for 2 topics (C1. International travel and testing, C2. Mask-wearing), Week 2 for 2 (C3. Scenarios and measures, C4. Seasons and restrictions), Week 3 for 2 (C5. Financial help, C6. Christmas and the new variant) and Week 4 for 1 topic (C7. Restrictions and New Year). Topics C8 to C11 (Lockdowns and restrictions, School closing, PCR tests, home office) didn’t vary significantly. In the group “Advice and communication”, most topics where more represented during either or both of the first two weeks (Topics D1. Prime minister statements, D2. Health secretary statements, D4. First minister statements), while Topic D3 (SAGE recommendations) was as much represented at Week 1 and Week 3 (non-significant change at Week 4), and Topics D5 to D7 (Irish government heath communication, communications about Northen Ireland, Declarations and social media) didn’t feature change during the four weeks. In the group “Foreign countries and regions”, two topics featured lesser representation at Week 2 with higher values the week before and after (E1. USA, E2 Europe), while the other topics didn’t feature significant variation (Topics E3. Asia, E4. Australia). About half of the topics in the group "Domains and events”, didn’t feature significant variation between the week (F6. Economy and inflation, F7. Christmas party cancellations, F8. sports, F9. Christmas, the coronavirus and the economy). Most of the remaining topics, featured their highest values in Weeks 3 and 4 (F2 to F5, relating to Politics, NHS and patients, Retail sales and Airlines), while topic F1 (market and investing) had its highest representation during Weeks 1 and 2. The remaining topics were not much interpretable or were related to durations. Most of these didn’t feature variation between the weeks.

## Discussion

The apparition and spread of the Omicron variant has been a turning point in the COVID-19 pandemic. In this contribution, we have examined important topics emerging from UK press articles reporting on Omicron, from the moment it was discovered until after it was announced the dominant variant in all regions of the UK [38]. We note that the need to make sense of the Omicron variant might have been particularly pressing to the population in the UK, which had been living under close to normal circumstances for a few months, after most COVID-19 restrictions had been removed in the summer—in contrast to many other countries. We have also analyzed the evolution of these topics during the considered period.

### Principal findings of the study

We have found that the mention of the origin of the variant was a device present in the media, which gradually decreased as time elapsed and the local and immediate threat of Omicron was more tangible to the public (see [15]) while other topics, related to less distant considerations increased in representation. The initial decline in restrictions on international travel, testing and the origins of Omicron and description of the virus was accompanied bya momentary increase in the representation of vaccination topics (except vaccination around the world), a more efficient means of protection for a circulating virus. This was concomitant with a rapid increase in the representation of infections, whereas the increase in the representation of the difficulties faced by hospitals and schools and business support was more delayed. In other words, the discussion in the press of pressing local matters took precedence over more distant ones after Omicron spread exponentially in the UK, and the risk was then presented as tangible and relevant to local life, rather than distant as in the first days of press coverage.

### Strengths and weaknesses in relation to other studies and differences in results

Our study examined for the first time the emergence and evolution of the press representation of Omicron, the SARS-Cov2 variant that, combined with broad vaccination, allowed society to recover from the pandemic. Prior studies [e.g., 16] focusing on COVID-19 mentioned *othering* as a reassuring mechanism used in the press when discussing emerging infectious diseases in far flung countries, allowing “people [to] ascrib[e] to particular ideas that construe the in-group as immune from threat and thereby afford collective symbolic coping” ([16], p. 968). As long as the disease is distant, othering allows the readership to feel safe and protected (“not me, not my group”; [15], p. vii). But as the disease progresses, and penetrates national boundaries, this is no longer a sustainable view of the situation. Changes in the early understanding of COVID-19 were investigated with a focus on othering in the press in Italy [13] as well, indicating a change of the conception of the risk, from distant to proximal. The question of othering was also investigated in relation to propagation and prevention of prejudice to the Chinese population (e.g., China virus) in newspapers published in the UK, the US and China [28]. In our study, even though blaming a culture with allegedly less hygienic practices for the disease could not be psychologically protective in the context of Omicron, because previous variants had spread in the UK (and it was a matter of time before Omicron did), the pattern of mentioning distant origins at the beginning and less so later on was apparent in our data.

The manual approach of [27] allowed them to identify criticism of the policies in place, as well as patterns of causal attribution and moral evaluation which are not transparent in a topic modeling study. A study examined the transgenerational divide in a visual analysis of press photographs [29]. In comparison to their study, ours which investigated only texts, was not aimed at illustrating the divide between groups, and our automated approach would not have been of much help for this research question. We note that, interestingly, the divide observed by [29] corresponds to the differentiated risks faced by the different generations (lowest in Children and highest in the elderly).

In their qualitative analysis of the press on COVID-19, [28] have shown that there was a racialistion of the pandemic. Although the origins of Omicron were an important topic in our study, our findings are not capable of helping us identify whether prejudice or the avoidance of prejudice of South Africans was frequently mentioned in relation to the origins of the virus. The question of racial prejudice is also an issue that our approach is not equipped to handle systematically.

The question of risk seems to be a common denominator in these articles. Our study is no different, as the themes that emerged from the topic modeling analyses we performed on close to 1500 UK press articles notably highlight notions related to questions such as “What are the risks?” (see the topic group “Domains and events”, with topics such as sales and the economy, or patient care), and the topics focused on infection and cases in the topic group “Symptoms, cases and vaccines”), “Who is at risk?” (emergence in South Africa vs local risk), “How to minimize the risk?”—see the topic group “Restrictions and measures” and the topics related to (booster) vaccination in topic group “Symptoms, cases and vaccines”. These findings share similarities with those of [27] in the Australia press and those of and [13] in Italy in relation to the emergence of the representation of the coronavirus in general.

## Conclusion

Overall, our study examined an original object of press representation, Omicron, the SARS-Cov2 variant that, combined with broad vaccination, allowed society to recover from the pandemic. The press is the carrier of the public discourse, unlike the textual productions of individuals [53, 54].

In the social sciences in general, there has been very limited research interest on Omicron. It is important to examine the representation of a variant from the perspective of the social researcher, as much as it is from the perspective of the biologist or the virologist, because part of the existing knowledge can apply, and part might not. Such investigation should be carried out in priority in regions featuring the first important clusters of cases, as we did in this study (the UK). This is a major theoretical implication of our study, as nothing is otherwise known of the topics of the press representation of Omicron.

We relied upon topic models for this investigation, an approach that is immensely valuable as it affords the discovery of patterns of change in sensemaking in the news quickly, which is relevant for policy makers notably.

Our findings have practical implications for policy-making, as public officials relying upon information stemming from these methods in the future could examine in a matter of days the evolution of sensemaking in the press over short (weeks) or long periods (years) of time, which would allow them adapt their communication strategy (e.g., which of their points has been picked up by the press and which has not). An increase in the topic of infections or other topics relevant for their tasks might alert them of urgent matters they might not be aware of, without requireing them reading all journalistic pieces, or their summary, individually.

In the case of the emergence of the representation of Omicron in the UK, we note the evolution of sensemaking closely matched the changes in the public officials’ discourse relating to the pandemic, reflecting the trajectory of the infection, which was largely limited to foreign nations in the first few days (from November 24) with associated precautionary measures (e.g., border closing, testing of incoming travelers) to the dominance of the variant in the UK by the end of the year and its local consequences (e.g., overwhelming hospitals; the Prime Minister’s Plan B).

## Data Availability

We made data for this contribution available on osf.io: https://osf.io/ft9vm.

## Abbreviations

ANOVA: Analysis of variance
ASCII: American Standard Code for Information Interchange
COVID-19: Coronavirus Disease 19
DTM: Document term matrix
HSD: Honest significant difference
LDA: Latent Dirichlet allocation
NHS: National Health Services
SARS: Severe acute respiratory syndrome
SARS-CoV2: Severe acute respiratory syndrome coronavirus 2
SE: Standard error
PM: Prime Minister
UK: United Kingdom
URL: Uniform Resource Locator

## Footnotes

1 https://en.wikipedia.org/wiki/List_of_newspapers_in_the_United_Kingdom (accessed on 9th January 2022).
